# Lipophilic Constituents of *Rumex vesicarius* L. and *Rumex dentatus* L.

**DOI:** 10.3390/antiox2030167

**Published:** 2013-09-16

**Authors:** Mona A. Abou Elfotoh, Khaled A. Shams, Kevin P. Anthony, Abdelaaty A. Shahat, Magda T. Ibrahim, Nevein M. Abdelhady, Nahla S. Abdel Azim, Faiza M. Hammouda, Mostafa M. El-Missiry, Mahmoud A. Saleh

**Affiliations:** 1 Department of Phytochemistry, National Research Center, 12311 Dokki, Cairo, Egypt; E-Mails: monaaboelfetoh_nrc@yahoo.com (M.A.A.E.); khaledashams@yahoo.com (K.A.S.); aashahat@hotmail.com (A.A.S.); nahlaabdelazim@yahoo.com (N.S.A.A.); fmhammouda@yahoo.com (F.M.H.); missirym@yahoo.com (M.M.E.-M.); 2 Department of Chemistry, Texas Southern University, Houston, TX 77004, USA; E-Mail: anthonykp@tsu.edu; 3 Department of Pharmacognosy, Faculty of Pharmacy, Al-Azhar University (Girls), Cairo, Egypt; E-Mails: nevein_abdelhady@yahoo.com (N.M.A.); magda.tohamy_1963@windowslive.com (M.T.I.)

**Keywords:** antioxidants, omega fatty acids, Egypt, flavonoids, quinones

## Abstract

*Rumex dentatus* L. and *Rumex vesicarius* L., of the family Polygonaceae, are edible herbs growing wild in Egypt. Their lipoid constituents were examined by both liquid chromatography/mass spectrometry (LC/MS) and by gas chromatography/mass spectrometry (GC/MS). Their essential oil compositions consisted mainly of thujene, limonene, fenchon, estragole, and anethole but at largely different concentration. Fatty acid compositions were similar among the two species and consisting of palmitic, oleic, linoleic and linolenic acids, with *R. vesicarius* containing much higher level of omega-3-fatty acids. Both of the crude lipid extracts of the two species showed strong antioxidant activity as a radical quenching agent against 2,2-diphenyl-1-picrylhydrazyl (DPPH) systems. Antioxidant activities were mostly associated with the polar lipid fractions. High performance thin layer chromatography (HPTLC), both in the normal and reversed phase, as well as liquid chromatography/mass spectrometry (LC/MS) in the positive and negative electrospray ionization (ESI), showed unique chemical profile for each species that can be useful for species identification and quality control of herbal drug formulations. *R. vesicarius* was characterized by abundances of flavonoids and *R. dentatus* was abundant in anthraquinones and chromones.

## 1. Introduction

*Rumex vesicarius* L. and *Rumex dentatus* L., of the family Polygonaceae, are the most abundant of the nine known *Rumex* species growing wild in Egypt [1]. They have been traditionally used in Egypt as medicinal herbs. *R. vesicarius* is an edible weed, eaten fresh or cooked, and commonly known in Arabic as “Humaidah” and in English as “Bladder dock”. As a medicinal herb, it is used in treatment of liver diseases, digestive problems, toothache, nausea, pain, anti-inflammatory, antitumor as well as antischistosomal, and antimicrobial activities [2,3,4,5,6]. It was also found to have aphrodisiac effect [7,8]. *R. dentatus* is known as toothed dock. Traditionally it is used as bactericidal, anti-inflammatory, antitumor, anthelmintic, astringent, and anti-dermatitis, in addition, its roots are also used in folk medicine for treating acariasis, eczema, diarrhea, and constipation [5]. *R. dentatus* extracts showed significant antioxidant activity [9].

Few phytochemical studies are found in the literature describing the chemical constituents of the two *Rumex* species described here. Most of them were carried out on the aqueous or alcoholic extracts of leaves, roots, and fruits of the plants, but none investigated the lipophilic constituents of the two species. In this manuscript we examined the lipophilic constituents as well as the chemical profile of the two species using different chromatographic and spectroscopic techniques.

## 2. Experimental Section

### 2.1. Plant Materials

*Rumex vesicarius* L. was collected from Al-Fayoum Governorate in Egypt, during March, 2010. *Rumex dentatus* L. was collected from Al-Sharqia Governorate in Egypt, during April, 2010. The two plant species were identified by Professor Kamal Zayed, Botany Department, Cairo University. A voucher specimen was kept in the herbarium of the National Research Center of Egypt. Aerial parts of the plant species were air dried and ground into fine powder.

### 2.2. Extraction and Fractionation

**Figure 1:**
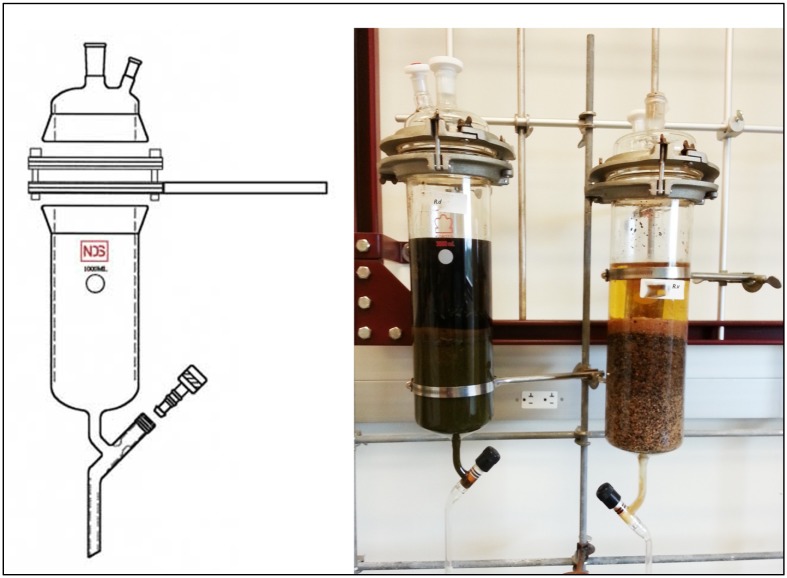
Percolator extractors used for solvent extraction of plant materials.

The air dried powder of each plant species (10 g) were, separately, extracted at room temperature for 48 h in percolators (shown in Figure 1) using 150 mL chloroform/methanol (2:1, by volume) as described by McCloud, 2010 [10]. The process was repeated three times and the combined extracts were evaporated at reduced pressure using a rotary evaporator at a temperature below 50 °C. Residual brown extracts were kept under argon at −20 °C until further analysis. Extracted yield was 0.8578 and 0.8313 g for *R. vesicarius* L. and *R. dentatus* L., respectively.

### 2.3. Essential Oils Preparation

Three 35 g replicate of each sample were subjected to hydrodistillation in a Clevenger-type apparatus according to the European Pharmacopoeia [11] until there was no significant increase in the volume of oil collected. The oil phase was separated, dried over anhydrous sodium sulfate, and kept in a dark glass bottle at 4 °C until the analysis.

### 2.4. Gas Chromatographic-Mass Spectral (GC/MS) Analysis of Essential Oils

GC/MS analysis of the essential oil was carried out using an HP5890 Series II Gas Chromatograph, HP 5972 Mass Selective Detector and Agilent 6890 Series Autosampler (Agilent Technologies, Santa Clara, CA, USA). A Supelco MDN-5S, 30 m × 0.25 mm capillary column with a 0.5 µm film thickness was used with helium as the carrier gas at a flow rate of 1.0 mL/min. The GC oven temperature was programmed at an initial temperature of 40 °C for 5 min, then heated up to 140 °C at 5 °C/min and held at 140 °C for 5 min, then heated to 280 °C at 9 °C/min and held for five additional minutes. Injector and detector temperatures were set at 250 °C. Mass spectrometry was run in the electron impact mode (EI) at 70 eV. The identification of the chemical constituents of the oil was determined by their GC retention times (tR), retention indices (KI) and interpretation of their mass spectra and confirmed by mass spectral library search using the National Institute of Standards and Technology (NIST) database with those of authentic samples or published mass spectral data [12]. The retention indices were calculated for all of the volatile constituents using a homologous series of C_8_–C_20_ *n*-alkanes. The quantitative data were expressed as relative percentage of the oil constituents calculated from the GC peak areas without using correction factors, and each oil was analyzed three times.

### 2.5. GC/MS of Fatty Acids Methyl Esters (FAMES)

50 mg of each sample extract was dissolved in 2 mL of 1.5% H_2_SO_4_ in methanol, vortexed, and left for 3 h in a heating box at 90 °C. The hydrolysis reaction was stopped by the addition of 2 mL water to each tube. The fatty acids were then extracted by shaking the aqueous layer with 5 mL of hexane, then passing the hexane layer over anhydrous sodium sulfate to remove water traces. The concentrated hexane layers were analyzed by GC/MS. GC/MS was carried out using the instrument described above. The GC oven temperature was programmed at an initial temperature of 130 °C for 1 min, then heated up to 300 °C at 5 °C/min and held at 300 °C for 5 min. Injector and detector temperatures were set at 250 °C. Mass spectrometry was run in the electron impact (EI) at 70 eV. The identification of the chemical constituents were determined by their GC retention times, interpretation of their mass spectra and confirmed by mass spectral library search using the NIST database.

### 2.6. High Performance Thin Layer Chromatography (HPTLC)

All HPTLC experiments were performed using the CAMAG automatic system programmed through WIN CATS software. Half gram of each sample extract was dissolved in 10 mL chloroform and 2 g anhydrous sodium sulfate was added, vortexed, and filtered. 100 µL of each chloroform extract was diluted to 1 mL and subjected to HPTLC under the following conditions:

Solutions of samples (equivalent to 50 µg of oil) were applied on HPTLC Nano-silica gel 60 F254 (10 × 10 cm) glass plates (Sorbent Technologies Inc., Norcross, GA, USA). Each sample was applied as a 10 mm band onto the plate using a CAMAG Automatic TLC Sampler 4 System and developed in a CAMAG automatic developing chamber (ADC2) with developing solvent of petroleum ether/diethyl ether/formic acid (90:10:2 by volume) for separation of nonpolar compounds. In a separate experiment, another developing solvent was used (toluene/ethyl acetate 7:3, by volume) for separation of relatively polar compounds. After drying the plates, they were viewed under ultraviolet light at wavelengths of 254 nm and 366 nm, and documented by photography. Vanillin/sulfuric reagent solution was used for the derivatization of the plates, which was prepared by adding 0.7 g of vanillin to 196 mL of methanol and 7 mL of sulfuric acid. The derivatization reagent was added to the tank designed for the CAMAG Immersion Device III. The plates were then attached to the device and lowered into the solution for 3 s, removed, and dried. The plates were then heated for 5 min in the oven at 110 °C until optimal colorization was observed. After cooling the plates, pictures were taken under 366 nm and white light to record the results. The documentation of the TLC plates was carried using the CAMAG Reprostar 3 System equipped with a DXA252 camera with a 16 mm lens. Plates were also derivatized in CuSO_4_ reagent (prepared by dissolving 20 g of copper sulfate pentahydrate in 200 mL methanol at less than 20 °C, then, under cooling with ice, 8 mL of 98% sulfuric acid and 8 mL of 85% orthophosphoric acid were added). The plates were dipped in the copper sulfate reagent for 5 s and allowed to dry for 1 min then heated for 30 min at 140 °C. Reverse phase HPTLC was carried out using 10 × 10 cm C_18_ silica HPTLC plates w/uv 100% silanization, glass backed 200 µ and developed with dichloromethane/acetic acid/acetone (20:40:50, by volume). Plates were also derivatized in 1% vanillin reagent, (1 g of vanillin, 100 mL of ethanol mixed with 5 mL of concentrate sulfuric acid, and 95 mL of ethanol), and the plate was dried in the oven at 100 °C for 5 min, after which spots were visualized under white light and 366 nm. Densitometry scanning was done using TLC scanner III (CAMAG, Wilmington, NC, USA) in the absorbance mode at 296 nm.

Solutions of the essential oils in methanol were applied to silica gel plates in 1 cm bands at 20 µg/band. Plates were developed with toluene/ethyl acetate (95:5, by volume). Plates were dried and observed at a UVwavelength of 254 nm to detect spots that have the ability to absorb UV light, which appear as a dark spot with green background. UV light of 366 nm was used to detect spots with fluorescent ability. Plates were also derivatized in vanillin reagent and the plates were dried in the oven at 100 °C for 5 min, after which spots were visualized under white light and 366 nm wavelengths.

### 2.7. 2,2-Diphenyl-1-picrylhydrazyl (DPPH) TLC Bioautography

TLC plates were developed as described above but then dipped in a 0.2% methanolic DPPH for one second and kept in the dark for 30 min, then observed under white light using CAMAG Camera. Active spots were seen as yellow bands on a violet background.

### 2.8. High Performance Liquid Chromatography/Mass Spectrometry (HPLC/MS)

HPLC of the crude *Rumex* extracts were performed on Agilent 1100 HPLC using 250 mm × 4.6 mm C18 column with acetonitrile/water (40:60, by volume) as the mobile phase at a flow rate of 1 mL/min and detected at 288 nm. High Performance Liquid Chromatography and Mass Spectrometry (HPLC/MS) was used to determine the chemical composition of each the crude extracts. HPLC was performed on Agilent 1100 HPLC/MSD SL and VL using Phenomenex Kinetic 2.6 µC_18_100A100 × 4.16 mm column with atmospheric pressure chemical ionization (APCI) and electrospray (ES), respectively. Each system is composed of the following units: a solvent delivery module, an automatic sampler injector, (100-well capacity), a controller module, a column oven, and a model series 1100 MSD mass spectrometer. Methanol/water/formic acid (90:10:1, by volume) was used as mobile phase A and 0.1% formic acid for mobile phase B at a gradient flow rate of 0.5 mL/min. Solvent A = 55% 0.1 formic acid (CH_2_O_2_), solvent B = 45% MeOH/H_2_O/CH_2_O_2_ (90:10:1, by volume). Starting at time 0, 45% B, at 15 min, increased to 65% B, at 15.5 min decreased to 45% B and held at 45% B for 5 min, and the run ended after 20.5 min. Diodearray detection at 288 nm, mass spectroscopy was performed using single ion monitoring in the negative modeat ions 327.10 and 505.10, using quasi molecular ions [M − H]^−1^ with a dwell time of 294 ms. Nitrogen was used both as drying gas and nebulizing gas at flow rates of 12 mL/min) and 35 (psig) respectively. The temperature of the drying gas was set to 350 °C. Data collection was handled using Chemstation V.B.04.02.

## 3. Results and Discussion

### 3.1. GC/MS Analysis

**Figure 2:**
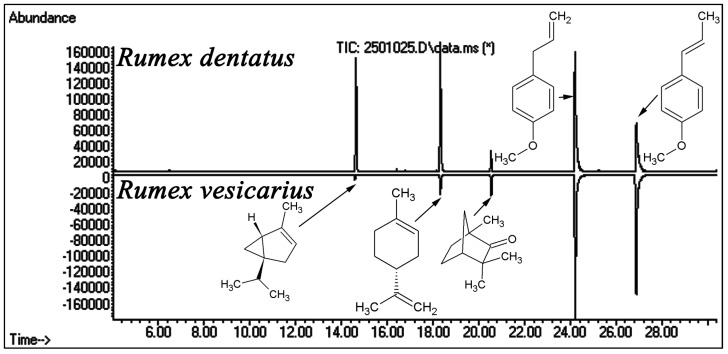
Total ion chromatograms (GC/MS) of the essential oil of *Rumex dentatus* and *Rumex vesicarius*. Compounds identification is shown in Table 1.

GC/MS analysis of the essential oil of the two *Rumex* species revealed five major monoterpenes, however, their concentrations in the two species were greatly different (Table 1). Total ion chromatograms of the essential oils of the two species and the chemical structures of the identified monoterpenes are shown in (Figure 2).

**Table 1:** Major monoterpenes identified and their percentage in the essential oil of *Rumex dentatus* and *Rumex vesicarius*.

Retention Time (min)	CompoundsIdentified	Kovatsindex *K*_I_	*R. dentatus* %	*R. vesicarius* %
14.64	α-Thujene	873	20.9	1.2
18.33	Limonene	1014	23.9	5.0
50.54	Fenchone	1121	3.7	5.1
24.21	Estragole	1178	34.7	46.9
26.89	Anethole	1279	16.9	41.8

**Figure 3:**
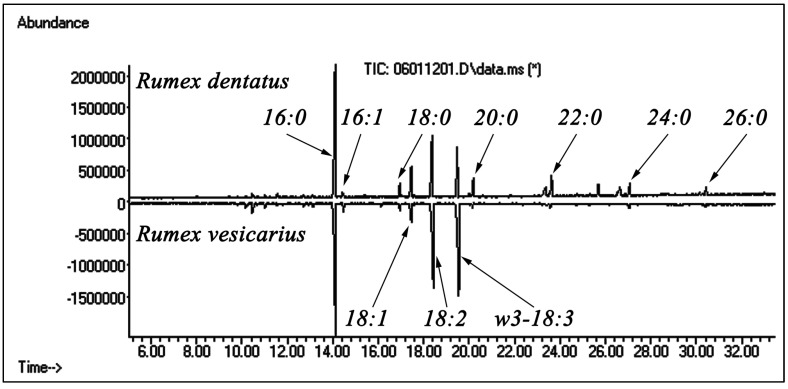
Total ion chromatograms (GC/MS) of fatty acid methyl esters of *Rumex*.

GC/MS analysis of the fatty acid methyl esters of the two species also showed significant difference between the two species although they were similar to some extent in their chemical composition but their percentages were different as seen in (Figure 3). Both species contain high level of palmitic acid, which is known to be found in all plants. Oleic and linoleic acids were also found in the two species. *Rumex vesicarius* was uniquely rich in the omega-3-fatty acids and *Rumex dentatus* showed the presence of C_24_ and C_26_ fatty acids where they were minor in the *R. vesicarius*. Table 2 presents the chemical composition of the fatty acids and related compounds in the two species.

**Table 2:** Fatty acids methyl esters composition of *Rumex dentatus* and *Rumex vesicarius*.

Retention	Percentage %		Identified Compounds
Time (min)	*Rumex dentatus*	*Rumex vesicarius*	
10.16	ND	1.5	Hydrocarbon C_13_H_22_
10.47	ND	2.7	C_11_H_20_O_2_
11.01	ND	1.4	C_20_H_40_O
11.55	1.0	1.0	Meristic Acid methyl ester
12.72	ND	1.0	12-methyl tetradecanoic acid
14.10	33.8	32.0	Palmitic acid methyl ester
14.44	1.7	1.5	7-C16:1
16.95	2.9	1.9	Stearic acid methyl ester
17.45	7.2	4.5	10-Oleic acid methyl ester
18.37	13.8	21.1	Linoleic acid
19.48	11.7	26.0	W3 linolinic acid methyl ester
20.19	4.0	1.0	C20:0 methyl ester
23.35	4.2	ND	Hydrocarbon C_23_H_48_
23.64	6.0	1.0	C22:0 methyl ester
25.69	3.7	ND	Squalin
26.65	4.3	ND	Hydrocarbon C_44_H_90_
27.07	3.2	0.8	C24:0 methyl ester
30.43	2.4	0.5	C26:0 methyl ester

### 3.2. HPTLC Analysis

**Figure 4:**
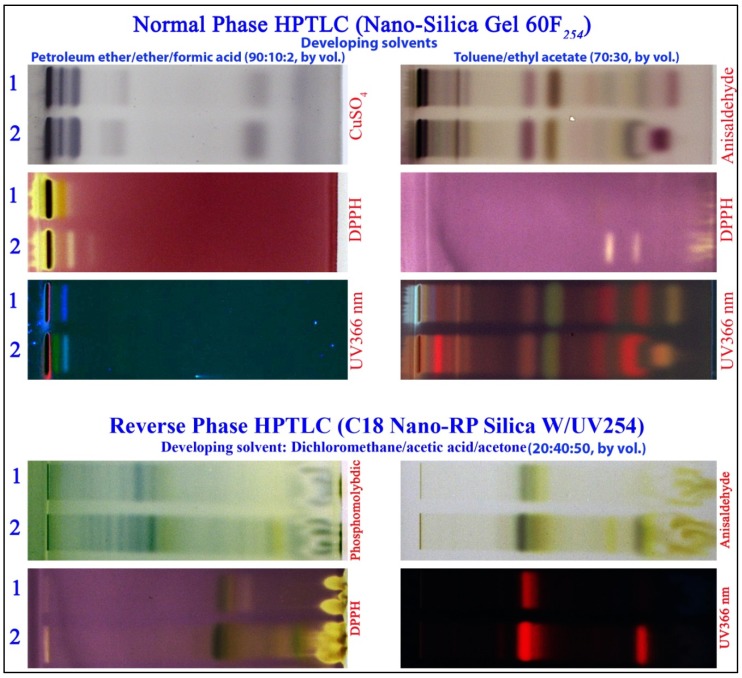
HPTLC profiles of *Rumex dentatus* (1) and *Rumex vesicarius* (2).

The HPTLC analysis of the chloroform/methanol extracts of *Rumex* species showed eightresolved bands when plates were developed in petroleum ether/diethyl ether/formic acid (90:10:2, by volume) and at least 10 resolved bands when using toluene/ethyl acetate (70:30, by volume) as developing solvent (Figure 4). Both species showed similarity in their compositions but each species has unique finger printing pattern that can be used to distinguish each sample by using copper sulfate and anisaldehyde as the derivatizing reagent. HPTLC finger printing is a valuable quality assessment tool for the evaluation of botanical materials, it allows for the analysis of a broad number of compounds both efficiently and cost effectively. The HPTLC method is simple, rapid, accurate, reproducible, selective and economic and can be used for quality control analysis and for quantitative determination of the plant metabolites. Reversed phase HPTLC of the two species revealed more differences among the two different species, as shown in Figure 4, using phosphomolybdic acid or anisaldehyde as derivatizing agent. The difference is clearly seen when plates were examined under UV light of 366 nm both for normal phase and reverse phase HPTLC, as shown in Figure 4. Bioautography experiment using DPPH radical quenching assay showed that *Rumex vesicarius* extract is stronger antioxidant than the *Rumex dentatus* extract, however, in both of them antioxidants were mostly found in the polar fractions (low *R*_f_ values near the origin in the normal phase and high *R*_f_ values near the solvent front in the reverse phase). This relative high antioxidant activity for the *Rumex vesicarius* is also consistent with the GC/MS analysis that showed higher level of omega 3-fatty acids and higher flavonoid content than those found in *Rumex dentatus*.

### 3.3. HPLC and LC/MS Analysis

**Figure 5:**
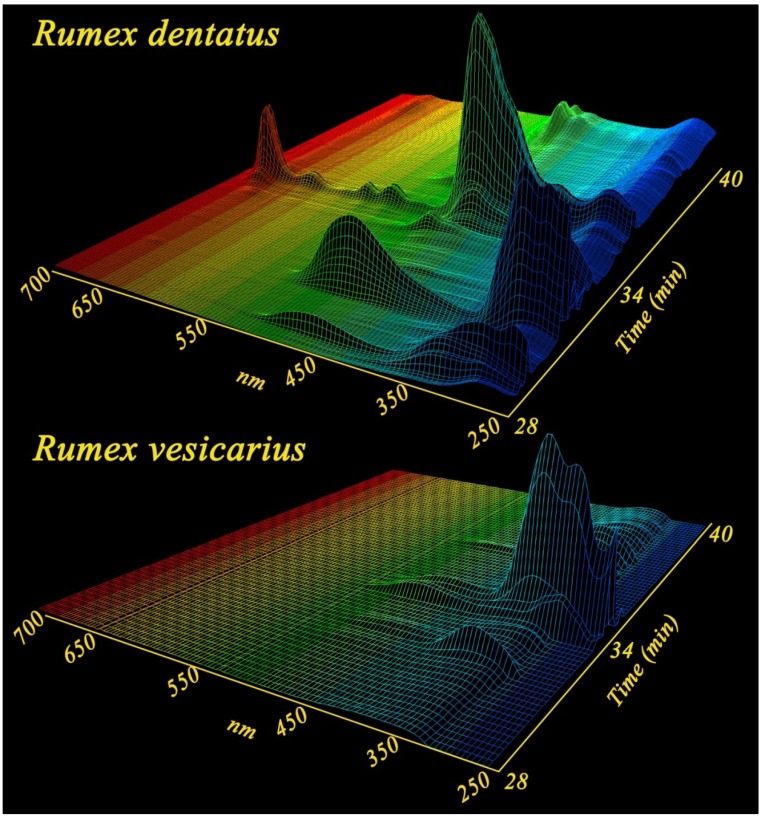
Ultra violet/visible absorption spectra LC chromatograms of *Rumex dentatus* and *Rumex vesicarius*.

**Figure 6:**
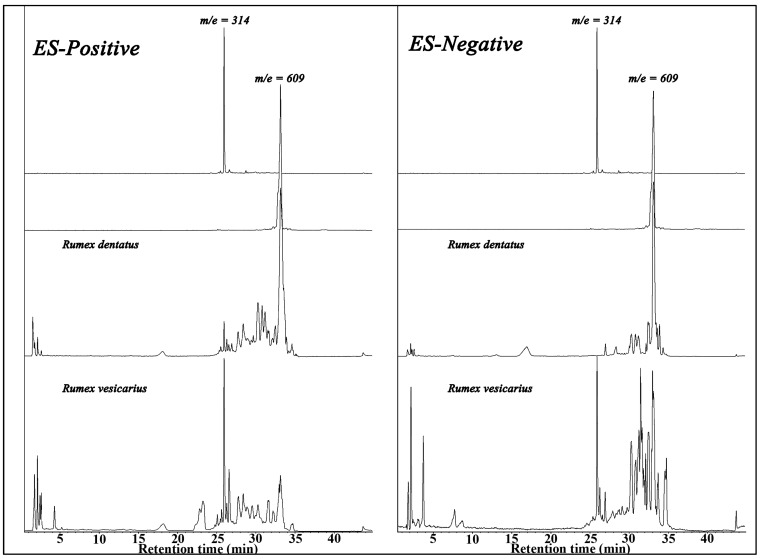
ESILCMS chromatograms of *Rumex dentatus* and *Rumex vesicarius*.

**Figure 7:**
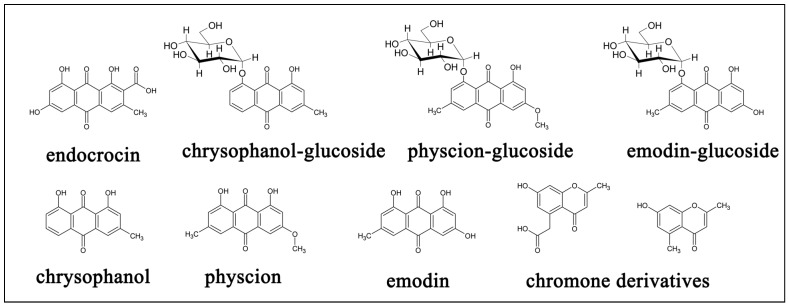
Chemical markers of *Rumex dentatus*.

**Figure 8:**
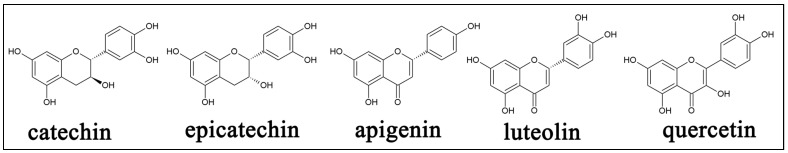
Chemical markers of *Rumex vesicarius*.

HPLC and LC/MS of the two species showed unique properties for both species with the diodarray detector (ultra violet and visible absorptions) as well as in the positive and negative total ion spectra. *R. dentatus* was characterized by the presence of high level of anthaquinones, chromones and naphthoquinones having distinguished UV absorbance at 450 nm as shown in Figure 5. On the other hand *R. vesicarius* is rich in flavonoides with a strong absorbance at 288 nm. Electrospray mass spectrometry of the two isomers both in the positive and negative modes as well as their retention times, relative percentage and base peak ions in their mass spectra are shown in (Table 3 and Table 4). ESI-MS negative mode was found to be more sensitive than positive mode for both species. Total ion chromatograms of the two species are shown in Figure 6. Several anthraqinones and chromones are characteristic markers for *R. dentatus* (Figure 7), however, flavonoids are characteristic markers for *R. vesicarius* (Figure 8) [13].

**Table 3:** Composition of the crude extracts of *Rumex dentatus* and *Rumex vesicarius* as analyzed by ESLCMS in the positive mode.

*Rumex dentatus*			*Rumex vesicarius*		
Retention time (min)	Percentage	Massspectrum	Retention time (min)	Percentage	Massspectrum
	%	basepeak(m/e)		%	basepeak(m/e)
1.68	1.0	144	1.79	1.8	130
2.09	1.2	144	2.13	5.7	130
2.25	0.6	144	3.70	4.1	130
2.53	0.7	144	7.69	2.3	120
16.86	3.9	197	25.88	6.8	314/528
16.94	2.5	197	26.23	2.1	558
26.95	1.7	277/353	26.91	1.8	353
28.32	2.8	181	27.88	2.1	279/360
28.74	0.6	277	29.09	2.0	149/277
29.69	0.6	279	29.74	1.4	314
29.98	1.3	353/537	30.27	7.6	277
30.28	5.3	277/537	30.82	5.4	279
30.77	4.6	279/496	31.22	6.8	277
31.15	5.5	277	31.43	6.9	332
31.62	1.1	279	31.64	3.9	149
32.14	1.3	496	31.80	1.7	344
32.39	4.7	279/496	31.91	1.3	360
32.52	3.6	537	32.09	2.4	360
32.69	0.6	593	32.48	7.8	279
33.08	45.3	609	32.85	1.8	545
33.41	2.9	593	32.96	5.2	404
33.55	2.6	480/474	33.09	7.7	593
33.85	3.9	488	33.58	1.0	460
34.30	1.6	516	33.67	2.2	478
			34.73	5.5	149/279

**Table 4:** Composition of the crude extracts of *Rumex dentatus* and *Rumex vesicarius* as analyzed by ESLCMS in the negative mode.

*Rumex dentatus*			*Rumex vesicarius*		
Retention Time (min)	Percentage	MassSpectrum	Retention Time (min)	Percentage	MassSpectrum
	%	Base Peak (m/e)		%	Base Peak (m/e)
1.51	2.3	104	1.72	4.1	130
1.75	0.6	130	2.10	3.6	130
2.11	0.8	130	2.42	1.8	120
18.09	0.9	197	2.59	1.9	144
25.50	0.7	544	4.28	1.6	120/142/326
25.93	1.7	314	18.21	2.2	197
26.27	1.1	275	22.80	4.4	433
26.52	0.9	277	23.21	5.9	433
26.90	1.1	277/492	25.06	1.3	241
27.72	3.8	275	25.61	1.5	528
28.37	5.9	181/291	25.93	11.5	314
28.85	1.0	275	26.27	2.3	275/351/625
28.95	2.1	275	26.55	5.7	528/488/625
29.38	1.9	275/518/699	27.77	6.2	275/625
29.65	1.8	518/627	28.37	6.8	181/291
30.25	9.0	277/553	28.86	4.6	275
30.77	5.9	279/496/	29.53	4.5	275/351
31.14	6.0	277/496	29.94	1.6	275/354
31.51	1.9	149	30.25	4.4	553
31.66	1.8	149	30.60	1.5	275
32.13	2.5	349/545/609	31.52	3.4	149
32.47	3.8	607	31.64	2.3	149
33.12	27.0	609	32.18	1.9	455
33.37	7.1	593	32.27	1.4	351/593
33.54	5.9	593	32.96	5.5	625
33.86	1.3	535	33.12	8.1	354
34.59	1.2	460	34.82		460

## 4. Conclusions

This study shows that lipoid fractions from aerial parts of *R. dentatus* and *R. vesicarius*, growing in Egypt, contained high levels of phenolic compounds, omega 3-fatty acids and exhibited significant antioxidant activities. Therefore, these phenolic-rich fractions may provide potential sources of natural antioxidants to be utilized in food and beverage products for inhibition of lipid oxidation. By comparing chemical profiles of the two species, we found that their chemical profiles showed significant difference. Our results may also be useful for quality control of rhubarb drugs, so as to guarantee their safe use in phytotherapies. On the basis of the data obtained in this study, *R. dentatus* leaves constitute a promising dietary source of biologically active compounds for the consumer, namely, phenolic compounds. In general, it may provide nutritional and health benefits associated with the consumption of fruits and vegetables. Thus, due to its agreeable taste and high phenolic content, it can be a good alternative in the preparation of salads.

The World Health Organization (WHO) has emphasized the need to ensure the quality of medicinal plant products by using modern controlled techniques and applying suitable standards. HPTLC is a simple, rapid and accurate method for analyzing plant metabolites. HPTLC fingerprint has better resolution and estimation of active constituents and is done with reasonable accuracy in a shorter time than HPLC or GC techniques. The HPTLC method can be used for phytochemical profiling of plants and quantification of compounds present in plants. With increasing demand for herbal products as medicines and cosmetics there is an urgent need for standardization of plant products. The chromatographic fingerprint is a rational option to meet the need for more effective and powerful quality assessment. The optimized chromatographic finger print is not only an alternative analytical tool for authentication, but also an approach to express the various patterns of chemical ingredients distributed in the herbal drugs and to preserve such “database” for further multifaceal sustainable studies. HPTLC finger print analysis has become the most potent tool for quality control of herbal medicines because of its simplicity and reliability. It can serve as a tool for identification, authentication, and quality control of herbal drugs.
